# A Two-stream Convolutional Network for Musculoskeletal and Neurological Disorders Prediction

**DOI:** 10.1007/s10916-022-01857-5

**Published:** 2022-10-06

**Authors:** Manli Zhu, Qianhui Men, Edmond S. L. Ho, Howard Leung, Hubert P. H. Shum

**Affiliations:** 1 Department of Computer and Information Sciences, Northumbria University, Newcastle upon Tyne, UK; 2 Department of Engineering Science, University of Oxford, Oxford, UK; 3 School of Computing Science, University of Glasgow, Glasgow, UK; 4 Department of Computer Science, City University of Hong Kong, Kowloon, Hong Kong; 5 Department of Computer Science, Durham University, Durham, UK

**Keywords:** Musculoskeletal disorders, Neurological disorders, Deep learning, Convolutional neural network, Feature fusion

## Abstract

Musculoskeletal and neurological disorders are the most common causes of walking problems among older people, and they often lead to diminished quality of life. Analyzing walking motion data manually requires trained professionals and the evaluations may not always be objective. To facilitate early diagnosis, recent deep learning-based methods have shown promising results for automated analysis, which can discover patterns that have not been found in traditional machine learning methods. We observe that existing work mostly applies deep learning on individual joint features such as the time series of joint positions. Due to the challenge of discovering inter-joint features such as the distance between feet (i.e. the stride width) from generally smaller-scale medical datasets, these methods usually perform sub-optimally. As a result, we propose a solution that explicitly takes both individual joint features and inter-joint features as input, relieving the system from the need of discovering more complicated features from small data. Due to the distinctive nature of the two types of features, we introduce a two-stream framework, with one stream learning from the time series of joint position and the other from the time series of relative joint displacement. We further develop a mid-layer fusion module to combine the discovered patterns in these two streams for diagnosis, which results in a complementary representation of the data for better prediction performance. We validate our system with a benchmark dataset of 3D skeleton motion that involves 45 patients with musculoskeletal and neurological disorders, and achieve a prediction accuracy of 95.56%, outperforming state-of-the-art methods.

## Introduction

Musculoskeletal and neurological disorders, such as joint problems, muscle weaknesses, and neurological defects (Table 1), are the most common causes of walking problems among older people, and they often lead to diminished quality of life. The prevalence of gait and balance abnormalities appears more than 60% in people aged over 80 years [1]. Gait analysis is a popular method for diagnosing these disorders. However, analyzing walking data manually requires trained professionals, and the evaluations may not always be objective [2]. We focus on proposing a low-cost automated tool for the early prediction and effective therapy monitoring of musculoskeletal and neurological disorders. First, it allows early intervention clinical care before the disorders develop into bigger health issues. Second, it supports clinicians make a more robust diagnosis by providing a computer-aided indicator and helps them effectively in monitoring patients’ health conditions.

Machine learning (ML) and deep learning (DL) have been widely used for automatically identifying health issues [3–7]. For example, by using 3D motion analysis, support vector machines (SVM) were applied for Parkinson’s disease classification in [8] from gait signals. Begg et al. [9] also classified young-old gait types with SVM from joint angle features. However, in the medical domain, such conventional approaches have restricted ability to model complicated data due to their limited capacity. They require considerable understanding and expertise for feature representation, i.e., feature engineering, since they have limited capability in processing raw data [10]. While deep learning allow multi-level abstractions of the raw data for decision making due to its deep architecture of non-linear hidden layers [11]. It facilitates the automatic diagnosis of disorders. While DL approaches are more advantageous with their deep hidden layer architectures. For instance, Davarzani et al. [12] used the long short-term memory (LSTM) network for human gait recognition from foot angle movements and achieved better performance than linear regression. McCay et al. [13] applied deep convolutional networks and achieved better prediction performance in cerebral palsy diagnosis than SVM, decision tree, and k-nearest neighbors algorithms (KNN).

Among different DL architectures, convolutional neural networks are very popular and have achieved promising performance on many diagnostic tasks [14–16]. However, these networks heavily rely on large datasets to avoid overfitting [17]. Unfortunately, large datasets are often not available in medical video/image analysis due to the restrictions on sharing data publicly in this domain. This makes it difficult to discover inter-joint correlations that are important for capturing coordination among different joints of human gait from raw joint features. We also observed that the majority of existing work only applies DL on individual joint features such as the time series of joint positions [13, 18]. As a consequence, these methods usually perform sub-optimally on smaller-scale medical datasets.

In this paper, we propose a two-stream CNN (2s-CNN) framework that explicitly takes both individual joint features and inter-joint features as input, allowing more effective discovery of features for disorder prediction from small data. The two different sets of features reflect different patterns of human motion data, i.e., the joint position describes the geometric location of an individual joint, and the relative joint displacement extracts the correlations of inter joints. To analyze the coordination and synchronization of different body part movements for better modelling walking motion, it is important to extract features from different joints simultaneously [7]. As such, we include the relative joint displacement feature which explicitly contains joint coordination patterns to guide the DL model to discover the essential inter-joint correlations for better diagnosis outcomes. To optimally model such distinctive feature, our network consists of two separate streams - a 3D joint position stream (3DJP-CNN) learning from the time series of joint position, and a 3D relative joint displacement stream (3DRJDP-CNN) learning from the time series of relative joint displacement. We further introduce a mid-layer fusion module fusing two single streams, which facilitates capturing both the individual joint information and inter-joint correlations, resulting in a more complementary representation of the data.

An extensive evaluation of the proposed framework is performed on the 3D skeletal motion dataset [19]. Different from [19], which evaluated the performance of a single type of feature on different off-the-shelf ML-based classifiers, our DL framework takes two types of features as input to model different aspects of the skeletal motion to achieve state-of-the-art performance. We further report the per-class classification performance to show the feasibility of deploying the framework as a patient diversion system, rather than only the overall average classification accuracy as in [19]. We justify our two-stream framework design by demonstrating its superior performance to the individual streams’ as a baseline study. To stimulate the research in this area, we open-source this project by releasing the source code for further validation and development. Our processed dataset features a standardized format, the evaluation protocol, as well as the augmented data for minimizing data bias. They can be downloaded at https://github.com/zhumanli/2s-CNN.

This paper is organized as follows. Data preparation is given in "Data Preparation" section. "Methodology" section presents the methodology. Experimental results and ablation studies are provided in "Experimental Results" section. "Conclusion" section concludes the research.

## Data preparation

Here, we explain the public benchmark dataset that we employ in this research ("The Dataset"), and our data augmentation strategy to deal with the data bias problem commonly found in medical datasets ("Data Augmentation").

### The Dataset

**Fig. 1:**
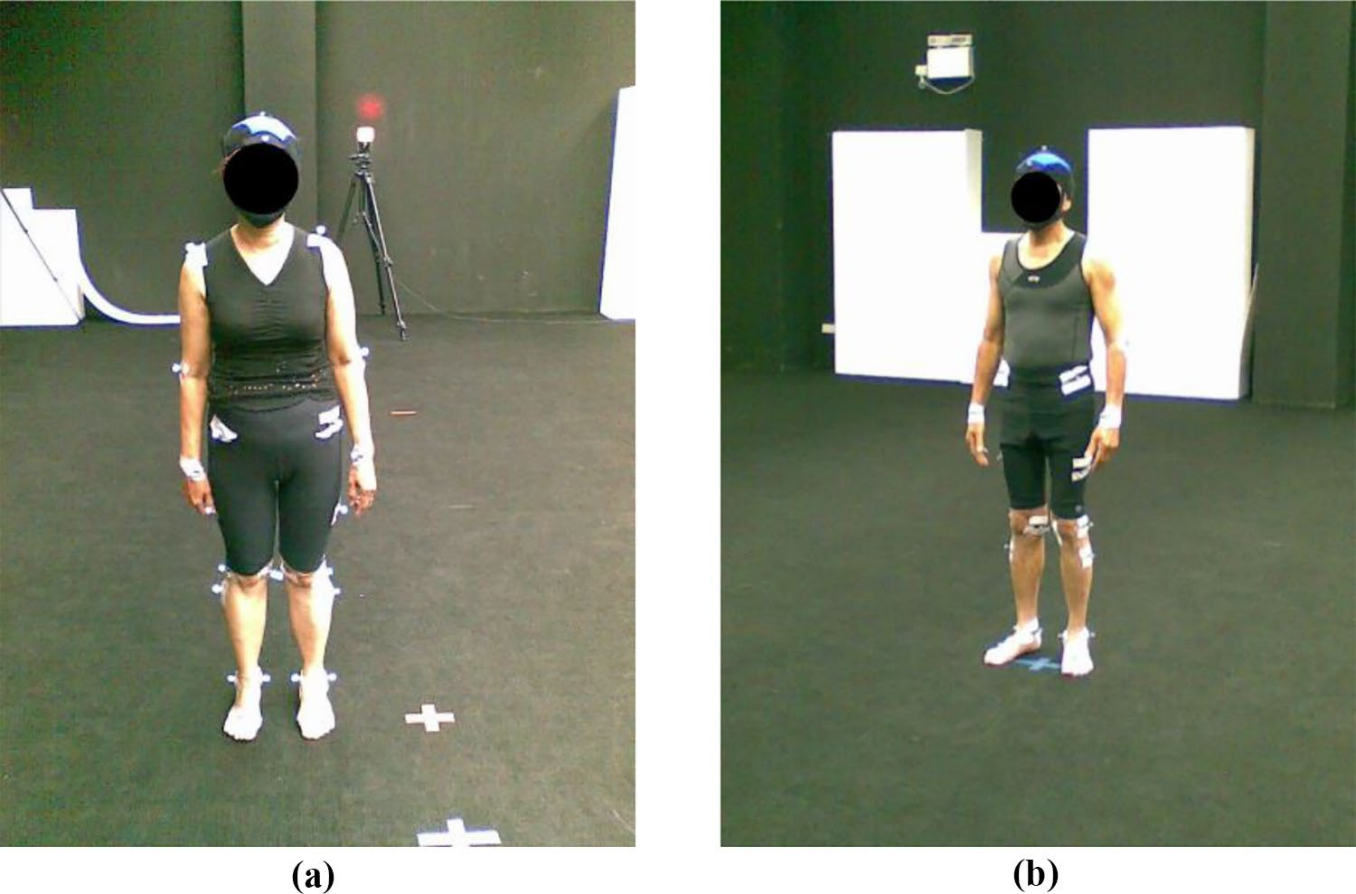
The optical motion capture system used with the Helen Hayes marker set structure illustrated in: (a) female example, (b) male example

We employ the dataset created by Rueangsirarak et al. [19]. It consists of 4 classes and 45 walking motions, i.e., 10 healthy, 4 joint problems, 18 muscle weakness, and 13 neurological defect motions. They were performed by 45 subjects, who were aged between 61 and 91 years old. The subjects were diagnosed to be one of the 4 classes by three medical doctors. The standard clinical test was used by medical experts for voluntary applicants’ screening and approval, e.g., applicants could walk without any assistance and had no other medical disorder history that could affect walking, and the details can be found in [19]. By applying a randomly sampled and population-based study, 5 male subjects and 40 female subjects were selected from the applicants’ approved list. The gender bias reflects the bias of the voluntary applicants in such a community. The data were captured using the Motion Analysis^®^ optical motion capture system [20] with fourteen Raptor-E optoelectronic cameras sampling at 100 Hz. The subjects were required to wear a motion capture suit, attached with a markers set on their body based on the Helen Hayes marker set structure [21], as shown in Fig 1. They were asked to walk naturally at their normal walking speed for 10 meters. The output of the motion capture system is 3D markers’ time-series positions. This optical marker-based capture method is advantageous because the captured data are more accurate than the markerless method, thereby facilitating more accurate diagnosis. Finally, the 3D positions of the joints are estimated from the marker locations by fitting a virtual character with similar body proportions to each subject in the software Autodesk MotionBuilder.

**Fig. 2:**
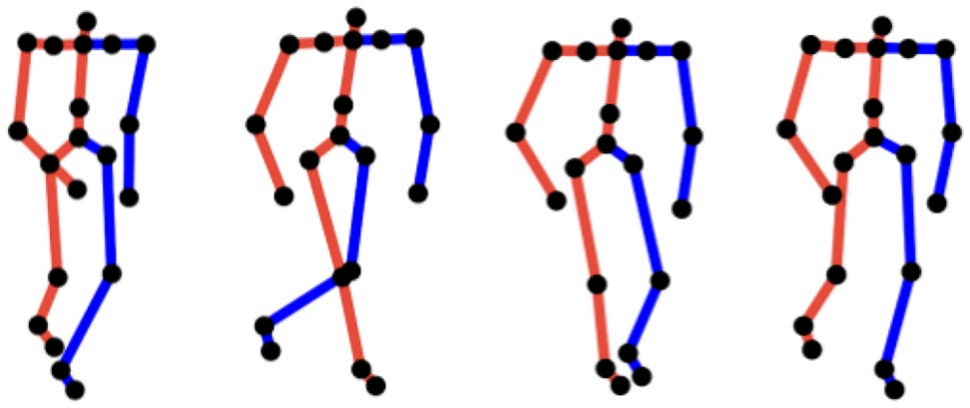
The sample of a human walking cycle (progressing from left to right)

**Fig. 3:**
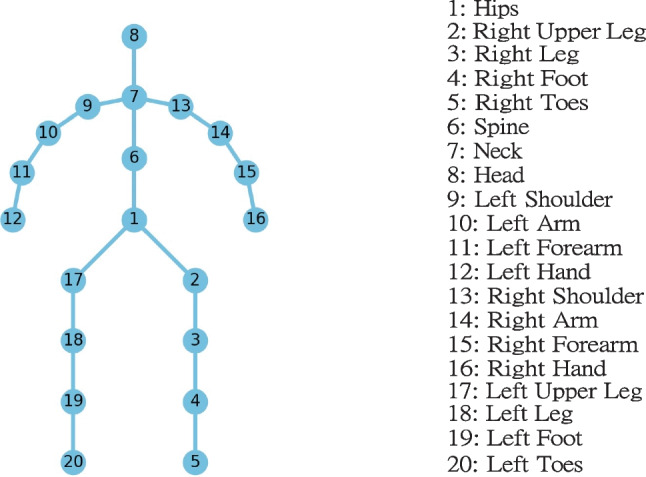
The overview of the skeleton structure

We perform normalization in both temporal and spatial domains using Autodesk MotionBuilder. For the temporal dimension, we extract three entire walking cycles of each subject from the raw data, and the intermediate cycle (includes the complete stance and swing phases) among three walking cycles is kept since it better represents the subjects’ normal walking motions. As the duration of each walking cycle is different, we temporally scale them using the linear interpolation [22] such that all motions will have the same duration. Fig. 2 illustrates a sample of a walking cycle. For the spatial dimension, the walking motion is normalized using rotation and translation operations such that the start positions and moving directions are the same among all motions and are comparable. Notice that end effectors such as end toes were removed as they are less informative and noisy. We then extracted 20 main joints that formed the final skeleton structure as shown in Fig. 3.

A major advantage of this dataset is that it includes multiple disorders, it facilitates the patient diversion system. Many existing computer-aided diagnostic systems of musculoskeletal and neurological disorders are limited to binary classification [23, 24], i.e., they simply differentiate between healthy and unhealthy data without specific types of disorders for unhealthy patients. With a model developed based on this multi-class dataset, patients can be transferred to a specific department as early as possible thus human resources can be greatly reduced.

### Data Augmentation

The used dataset in this study is challenging due to its multiple, small-scale, and biased classes of disorders. The number of training samples is a critical influencing factor on the generalization ability of DL models. To make the DL model generalize well, augmentation techniques such as random scaling, noise addition, sign inversion, and motion reverse were applied to generate more training samples in cerebral palsy prediction [25]. However, the aforementioned techniques only focus on intra-class variations. As a result, the augmented data may not be effective in alleviating the inter-class similarity problem as multiple class labels have to be considered. Here, we apply the synthetic data augmentation method *mixup* [26], resulting in an unbiased, 4 times larger dataset. This size is reasonable for our model learning, which prevents overfitting and generates good performance from our experiments.

*Mixup* [26] fits better to our dataset compared to data agumentation methods such as *Gaussian noise* [27] and *SMOTE* [28]. It generates synthetic data from real samples of different classes in an interpolation manner rather than adding random noise or generating within the same class. This facilitates the generation of reasonable synthetic data in scenarios when the data have specific structures (e.g., the hierarchical structure for body joint positions and angles on a human body) and limited samples are available.

The data augmentation operation is described as follows:

1 $$X_{v} = \lambda X_{a} + (1 - \lambda )X_{b}$$

where *X*_*v*_ is a synthetic sample, *X*_*a*_ and *X*_*b*_ are two samples randomly selected from class *a* and *b* respectively, and *λ ∈* [0*,* 1] represents how much contribution to the synthetic data from the two original ones. We empirically set *λ* = 0*.*9 based on the performance of our two-stream CNN framework, and the same label as *X*_*a*_ is assigned to the generated sample *X*_*v*_. By doing this, small inter-class similarities will be introduced which encourages the neural network to learn more discriminative deep representations to differentiate samples from different classes.

The augmentation is done for different cross-validations. Concretely, for each cross-validation, one fold is used for testing, and the remaining folds are firstly used to generate synthetic samples, then together with the generated synthetic samples are used for training. Besides presenting the number of samples of the original data, the statistic of data with augmentation is also given in Table 2.

**Table 1:** Class of disorders and examples

Class	Joint Problem	Muscle Weakness	Neurological Defect
	Sprains [29]	Muscular dystrophy [30]	Epilepsy [31]
Examples	Tendinitis [32]	Spinal muscular atrophy [33]	Alzheimer’s disease [34]
	Osteoarthritis [35]	Muscle fatigue [36]	Parkinson’s disease [37]

## Methodology

**Fig. 4:**
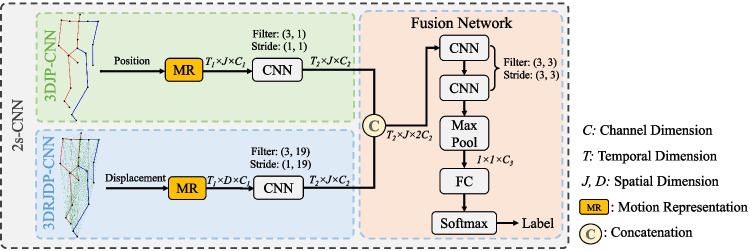
Overview of our proposed two-stream framework

We propose a two-stream convolutional neural network that explicitly takes two types of features as input, Fig. 4 illustrates its architecture. The 3DJP-CNN stream ("The Joint Position Stream") aims at modelling individual joint time series from joint positions, and the 3DRJDP-CNN stream ("The Relative Joint Displacement Stream") aims at extracting inter-joint correlations from relative joint displacements. The mid-layer fusion module ("The Two-stream Network and Feature Fusion") fuses the two single streams’ high-level output features to take advantage of both individual joint information and inter-joint correlations.

### The Joint Position Stream

**Fig. 5:**
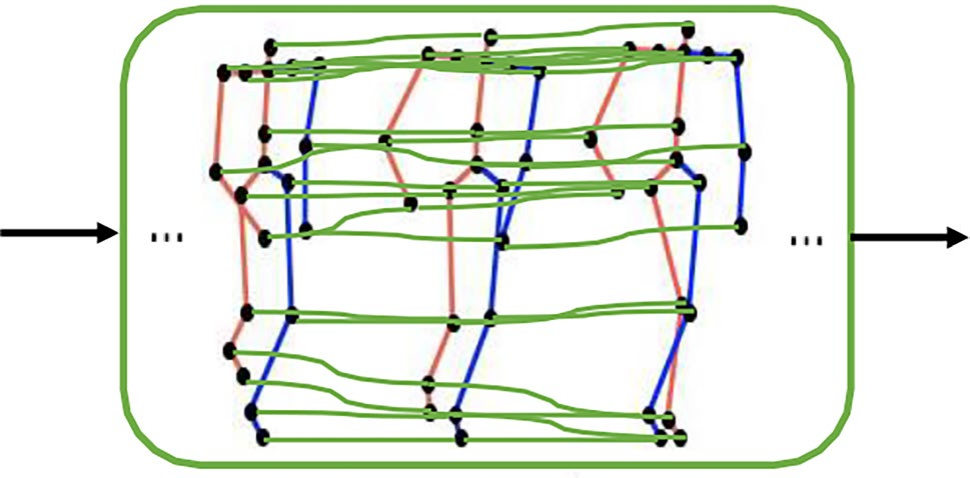
Modelling the time series of individual joint positions

As the first stream, we propose 3DJP-CNN that models the time series of individual joint positional information as shown in Fig. 5. To achieve this, the 3D coordinates are modelled as channels and a 2D convolution is applied on both temporal frame and spatial joint dimensions. Modelling coordinate dimension as a channel guarantees all three-dimensional coordinates can be covered at once by a filter rather than part of them to preserve the semantics.

More specifically, the input joint positions of this stream are represented as a feature tensor *S ∈ R*^*T* ×*J*×*C*^, in which *T* is the number of frames, *J* is the number of joints, and *C* = 3 represents the 3 coordinate dimensions of a joint position. Then, a 2D CNN layer is applied to obtain the extracted joint position feature *f*_*jp*_ = *Conv*2*d*(*S*). The filter in the convolutional layer of this stream has the dimension of *F*_*T*_ × *F*_*J*_ × *C*, where *F*_*T*_ = 3 and *F*_*J*_ = 1, and the strides are both set as 1 in our experiment. With this setting, the filter covers all coordinate dimensions at once and moves along the frame and joint dimensions. This ensures that the position information of every individual joint is encoded, and the local time-series information is modeled.

### The Rrelative Joint Displacement Stream

**Fig. 6:**
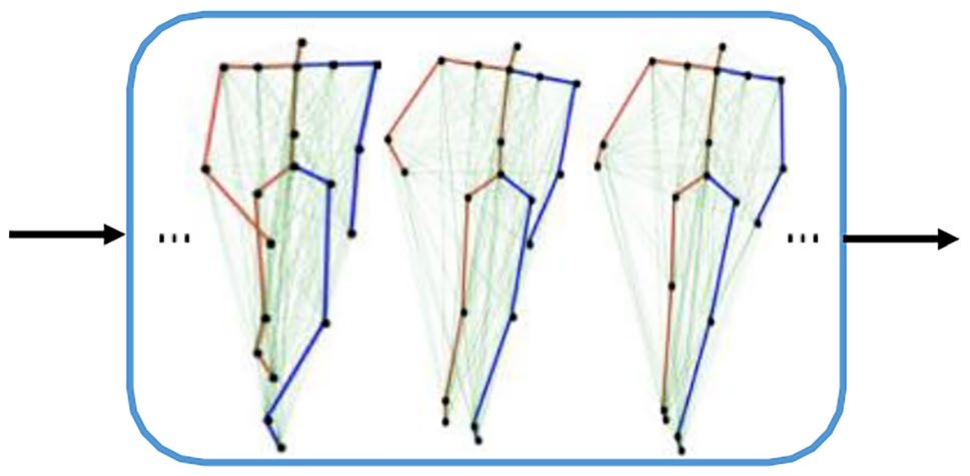
Modelling the inter-joint correlations over time

As the second stream, we propose 3DRJDP-CNN that models the correlations among different joint pairs over time as shown in Fig. 6. Compared with the commonly used individual joint position feature [38, 39], the joint-pair level feature 3DRJDP is more advantageous. Because it explicitly captures the inter-joint coordination patterns and provides more information to the network preventing overfitting of learning from a small dataset.

Concretely, given a skeleton sequence, the relative joint displacement is defined by calculating the displacement between all the joint pairs except pairs that connect to the same joint (i.e., self-loops), and it is represented as the set *Q* = *{D*_*t*_ (*i, j*) *| t* = 1*,* 2*, . . . , T* ; *i, j* = 1*,* 2*, . . . , J* ; *i ≠* *j}*, where *D*_*t*_ (*i, j*) denotes the relative displacement of joint *i* and *j* at time *t*:

2 $$D_{t} (i, j) = x^{i}_{t} - x^{j}_{t} , \;y^{i}_{t}- x^{j}_{t} , z^{i}_{t} - z^{j}_{t}$$

where $x^{i}_{t}$, $y^{i}_{t}$, and $z^{i}_{t}$ are the coordinates of joint i. Note that *D*_*t*_ (*i*, *j*) =  − *D*_*t*_ (*j*, *i*).

We represent the input of this stream as the feature tensor *S*^*′*^* ∈ R*^*T* ×*D*×*C*^, in which *D* is the number of *J* (*J −* 1) correlations since each joint has *J −* 1 correlations to all other joints. Then a 2D CNN layer is used to obtain the inter-joint feature *f*_*rjdp*_ = *Conv*2*d*(*S*^*′*^). The filter in this convolutional layer has the dimension of *F*_*T*_ × *F*_*D*_ × *C*, where *F*_*T*_ = 3 and *F*_*D*_ = *J −* 1, and the strides are set as 1 and *J −* 1 accordingly. Designing the spatial filter size as *J −*1 not only enables the network to extract the inter-joint correlations of each joint but also ensures it has the same output feature size as the first stream, which facilitates the mid-layer fusion of two single streams at the feature level.

### The Two-stream Network and Feature Fusion

We present a mid-layer fusion module that takes advantage of the 3DJP and 3DRJDP streams for better prediction performance. Different types of features usually reflect different data patterns, and the fusion of them can generate a more complementary representation, facilitating better prediction performance.

There are two main advantages of mid-layer fusion architecture. Firstly, it contains much richer information on the original data [40] and does not need to train multiple classifiers compared with the late-fusion. The follow-on CNN layers further facilitate adaptively adjusting and balancing the importance of different feature sets, resulting in better prediction outcomes. Secondly, compared with an early-fusion scheme (i.e., fusing at the first layer), the CNN layers in individual schemes help to extract useful, higher-level information from the raw features before fusion. It also ensures that the feature sizes in the two streams are compatible for fusion, since different raw feature sets are often incompatible with diverse sizes, different representation spaces of features, etc. We fuse the outputs of individual streams in their channel dimensions as shown in our framework (Fig. 4). In the first stream, each value of its output tensor represents the time series of an individual joint, and in the second stream, each value represents an inter-joint correlation. The fused feature tensor *f* = *concat*(*f*_*jp*_*, f*_*rjdp*_) takes the advantage from the two streams and has the dimension of *T*_2_ × *J* × 2*C*, it is then fed into two CNN layers for further process. After that, an adaptive Max Pooling layer is applied to extract the salient spatial-temporal information. Finally, a fully connected (FC) layer and a softmax layer are used to perform the classification.

The following cross-entropy loss function is applied for the evaluation of training and testing:

3 $$L =-\sum_{i=0}^{E-1}y_{i} \mathrm{log}\; (p_{i})$$

where *p* = [*p*_0_*, . . . , p*_*E−*1_] is a probability distribution, *p*_*i*_ represents the probability that a sample belongs to class *i*. *y* = [*y*_0_*, . . . , y*_*E−*1_] is the one-hot representation of class labels, and *E* is the number of classes.

## Experimental Results

In this section, we first compare our proposed two-stream approach 2s-CNN against ML-based methods. Then, we discuss the performance of the two-stream model based on every single stream. Finally, the ablation study is presented.

All the results are obtained from the proposed network implemented by PyTorch and trained in an end-to-end manner with Adam optimizer. The hyper-parameters *epoch*, *learning rate*, and *batch size* are set as 80, 0.003 and 57 respectively. Our evaluation is done by 5-fold cross-validation. The averaged outcome for all cross-validations is presented as the final result.

### Comparisons with the Other Methods

We compare our method with the start-of-the-art SVM approach with different kernels [19] and some other ML-based methods, including Decision Tree [19, 41], Bayes [42], and Random Forest [41]. We also compare with the fully connected deep network architecture FCNet that was designed in [13] for cerebral palsy prediction. To make a fair comparison, both 3DJP and 3DRJDP features are used in all these methods, and hyperparameters are turned to an optimal configuration in our experiment.

It can be seen in Table 3 that our proposed 2s-CNN achieves the best average accuracy of 95.56% on all classes, and the 3DRJDP-CNN outperforms 3DJP-CNN, FCNet, and ML-based methods. This demonstrates the power of convolutional neural networks and the advantage of fusing 3DJP and 3DRJDP features.

**Table 2:** The statistics of the original data and the augmented data

Class	Original data	With data augmentation
Healthy	10	45
Joint Problem	4	45
Muscle Weakness	18	45
Neurological Defect	13	45
Overall	45	180

### Comparisons with Baselines

We build two single-stream networks as baselines. The classification accuracy of our two-stream network and baselines is shown in Table 3 (the last three lines). We observe that 2s-CNN outperforms baselines and achieves a significant accuracy improvement of 4.4%. This demonstrates the two feature sets are complementary to each other under our fusion design. In addition, 3DRJDP-CNN performs on par with or better than 3DJP-CNN in all classes. We believe that this is because 3DRJDP carries explicit inter-joint correlations, which makes it more powerful in distinguishing healthy and unhealthy gaits.

**Fig. 7:**
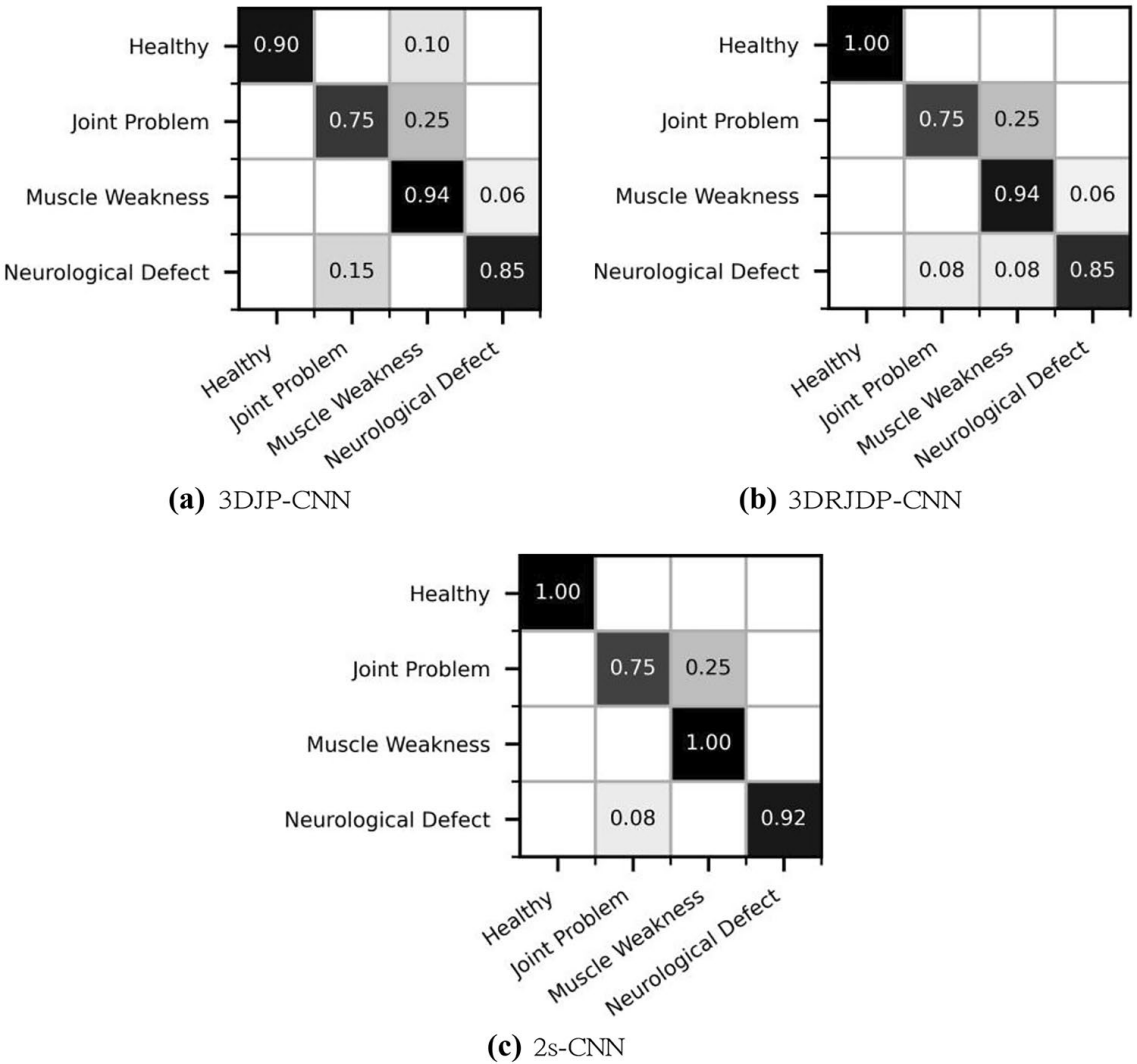
Confusion matrices of single-stream and the two-stream networks

To investigate the contributions of single networks and the improvement of the fusion module for different gait disorders and the healthy classes, Fig. 7 presents the confusion matrices. We observe that 3DJP-CNN fails to differentiate between healthy and unhealthy classes, and 3DRJDP suffers for distinguishing three unhealthy classes. We argue that this is because 3DJP-CNN does not contain explicit inter-joint correlations. More importantly, 2s-CNN takes advantage of 3DJP-CNN and 3DRJDP-CNN by only struggling in two unhealthy classes, i.e., joint problem and neurological defects. This indicates that the fusion module is having a positive impact on the overall classification performance.

**Fig. 8:**
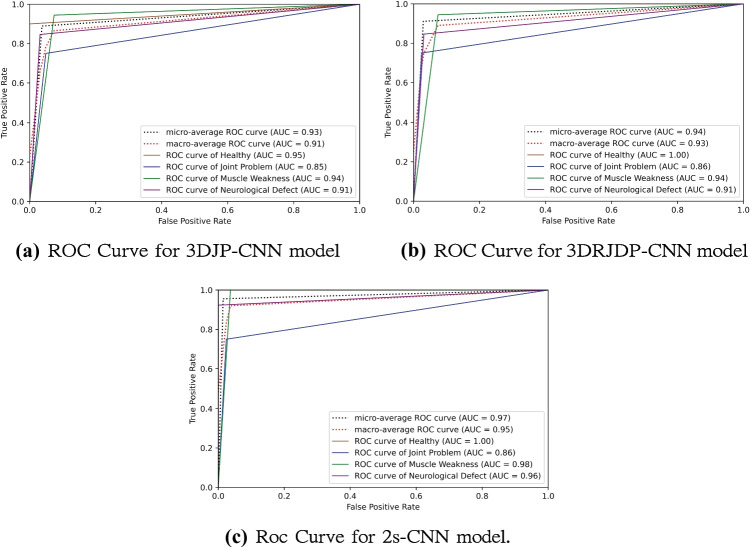
Receiver operating characteristic curves for multi-class disorder classification

To make a comprehensive comparison between the proposed two-stream network and baselines, besides accuracy, precision, recall, f1-measure, receiver operating characteristic (ROC) curves, and AUC (area under the receiver operating characteristic) are also reported in Table 4 and Fig. 8. The results show that our proposed 2s-CNN model achieves consistent superior performance on these measure metrics in both individual class and average evaluations.

**Table 3:** Comparisons with other methods in accuracy (%)

Method	Healthy	Joint Problem	Muscle Weakness	Neurological Defect	Average
Naive Bayes [28]	**100.00**	75.00	94.44	53.85	82.22
Random Forest [38]	**100.00**	75.0	83.33	**92.31**	88.89
Decision Tree [19, 38]	**100.00**	75.00	77.78	84.62	84.44
SVM (RBF) [19]	**100.00**	**100.00**	0.00	23.08	37.78
SVM (Linear) [19]	**100.00**	75.00	83.33	**92.31**	88.89
SVM (Polynomial) [19]	**100.00**	75.00	83.33	**92.31**	88.89
FCNet [13]	**100.00**	75.00	77.78	**92.31**	86.67
3DJP-CNN	90.00	75.00	94.44	84.62	88.89
3DRJDP-CNN	**100.00**	75.00	94.44	84.62	91.11
2s-CNN	**100.00**	75.00	**100.00**	**92.31**	**95.56**

The bold values represent the best accuracy for each class

**Fig. 9:**
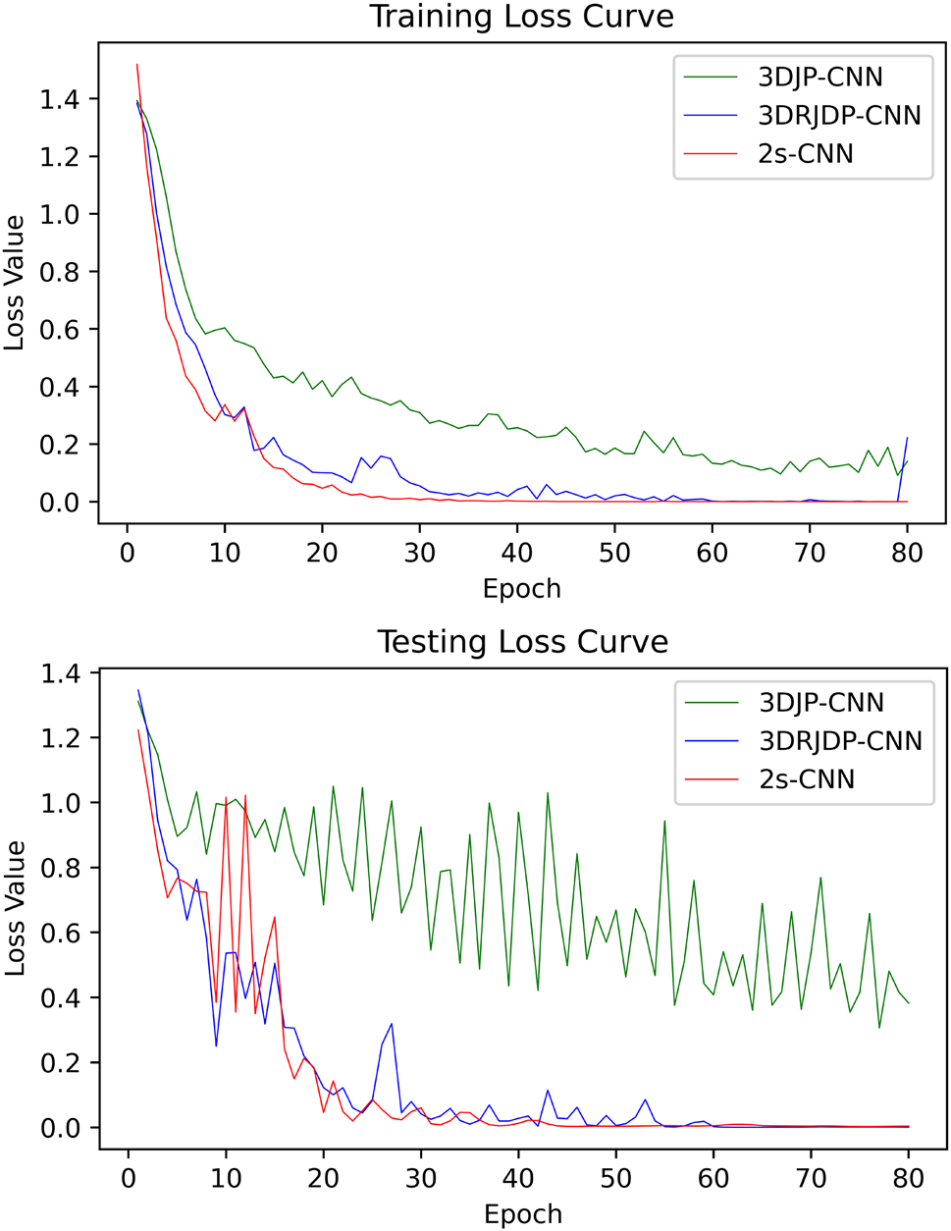
Training and testing loss curves of single-stream and two-stream networks

To further demonstrate the robustness of the proposed methods, their training and testing loss curves are presented in Fig. 9. It is can be seen that 2s-CNN generates more consistent and robust curves than 3DJP-CNN and 3DRJDP-CNN, and 3DRJDP-CNN performs better than 3DJP-CNN. This aligns with the results of the abovementioned evaluation results.

### Ablation Study

**Fig. 10:**
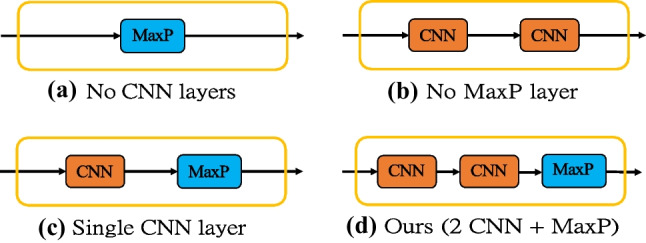
Proposed fusion network architecture with different CNN and MaxP combinations

To validate our network architecture, we compare our proposed fusion network architecture under different CNN and Max Pooling (MaxP) combinations as shown in Fig. 10. The number of parameters, averaged per-sample test time, and accuracy are reported in Table 5. We observe that the system only can achieve an accuracy of 91.11% without any CNN layers (No-CNN). The two-CNN architecture (No-MaxP) has no improvement compared with a single CNN layer (SinCNN), but it improves with the help of a Max Pooling layer (Ours). This is because two CNN layers have digested the majority of the features, and Max Pooling could further select the discriminative information. Notice that although our two-CNN architecture contains more parameters, the test time only demonstrates a slight increase.

**Table 4:** Comparison of results with baselines on precision, recall, f1-measure, and AUC

Metric	Network	Healthy	Joint Problem	MuscleWeakness	Neurological Defect	Average
	3DJP-CNN	**1.00**	0.60	0.89	0.92	0.85
Precision	3DRJDP-CNN	**1.00**	**0.75**	0.89	0.92	0.89
	2s-CNN	**1.00**	**0.75**	**0.95**	**1.00**	**0.92**
	3DJP-CNN	0.90	**0.75**	0.94	0.85	0.86
Recall	3DRJDP-CNN	**1.00**	**0.75**	0.94	0.85	0.89
	2s-CNN	**1.00**	**0.75**	**1.00**	**0.92**	**0.92**
	3DJP-CNN	0.95	0.67	0.92	0.88	0.85
F1-Measure	3DRJDP-CNN	**1.00**	**0.75**	0.92	0.88	0.89
	2s-CNN	**1.00**	**0.75**	**0.97**	**0.96**	**0.92**
	3DJP-CNN	0.95	0.85	0.94	0.91	0.91
AUC	3DRJDP-CNN	**1.00**	**0.86**	0.94	0.91	0.93
	2s-CNN	**1.00**	**0.86**	**0.98**	**0.96**	**0.95**

The bold values represent the best performance on each metric for each class

**Table 5:** The number of parameters, averaged per-sample test time, and accuracy of proposed fusion network architecture with different CNN and Max Pooling combinations

Method	#Params	Test Time	Accuracy
No CNN layers (No-CNN)	86,288	44.53 ms	91.11
No Max Pooling layer (No-MaxP)	238,864	**44.36 ms**	93.33
Single CNN layer (SinCNN)	**86,032**	46.94 ms	93.33
Ours (2s-CNN + MaxP)	233,488	47.29 ms	**95.56**

The bold values represent the best indicator of each metric

**Fig. 11:**
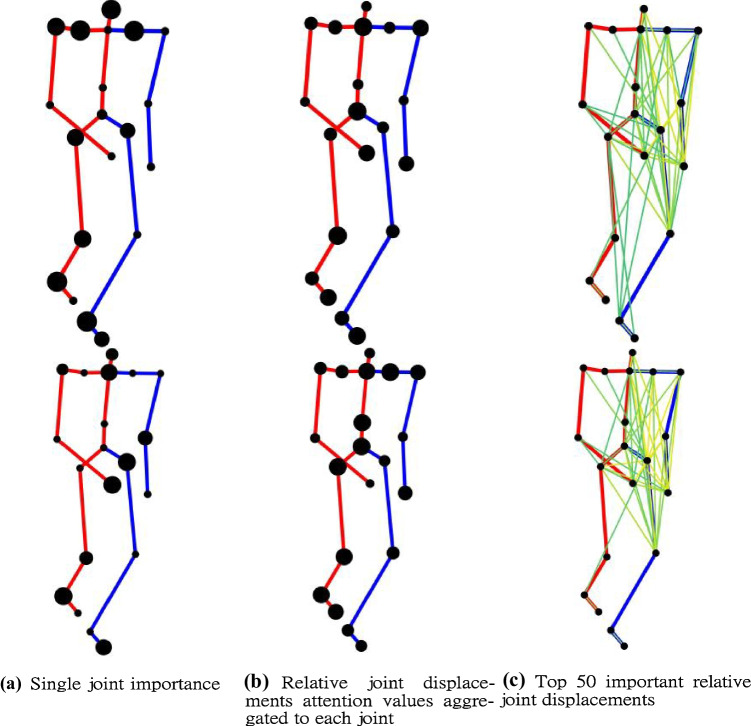
Visualization of the importance of different joints and relative joint displacements with a healthy sample (upper row) and a muscle weakness sample (lower row). The larger size of black joints represent higher importance. The relative joint displacements attention values are aggregated to each joint to show which joints have more interactions with other joints. The importance of relative joint displacements is visualized from a yellow to emerald green scale, with the yellow color representing higher importance

We further visualize the importance of joints and relative joint displacements across validations from 3DJP-CNN (with joint position features) and 3DRJDP-CNN (with relative joint displacement features) streams respectively using channel attention [43] as shown in Fig. 11. For single joint importance, we can see that the two streams mostly demonstrate different importance on the same joints, indicating that they focus on different aspects of a joint. Notice that they both have higher importance on the foot joints, this may be because the subject’s balance is impaired by walking issues, e.g., the foot/ankle proprioceptive input is decreased [44]. More importantly, the 3DRDJP-CNN stream assists in identifying which joint pairs are highly involved in the body movements, i.e., the left upper body joints tend to have more interactions. This could be because the subject tries to avoid using the right body parts due to pain, resulting in imbalanced gaits [45]. In addition, the body parts’ importance of the two samples shares similar patterns, indicating our model is consistent across classes. This kind of human-understanding visualization can effectively support clinicians for in-depth analysis, e.g., rapid lesion locating.

## Conclusion

In this paper, we have proposed a 2s-CNN framework that explicitly takes both individual joint features and inter-joint features as input for musculoskeletal and neurological disorders prediction. Our proposed mid-layer fusion module adaptively merges individual joint 3DJP and inter-joint 3DRJDP features into the network to jointly learn and update with the model, relieving the system from the need of discovering more complicated features from small data. The experimental results have shown that the inter-joint 3DRJDP features demonstrated more effectiveness for different disorders classification, which aligned with the intuition that movement is generated by body parts’ coordination. The method *mixup* [24] was used to deal with the data bias problem, resulting in a more robust system. We demonstrated the effectiveness of the mid-layer fusion of fusing the two sets of features. Compared with ML-based methods and the fully connected deep network, our proposed model outperforms them with a better average prediction accuracy of 95.56%. The accuracy of every individual class is also reported for the first time.

Interpreting DL models is critical in the medical area because it can provide us with more insights into these advanced automatic tools, thus gaining the trust of clinicians and patients. Our interpretable visualization of spatial attention facilitates a user to focus the analysis on the body parts with high attention. For future study, we intend to interpret the proposed framework from both spatial and temporal domains for frame-to-frame interpretation, to generate a more solid automatic musculoskeletal and neurological disorders prediction system. As the current dataset is relatively small, we will consider enlarging it in future works, e.g., increasing the number of subjects and the video length including walking cycles, such that other important factors (e.g., step-to-step variability) could be better included for a more robust diagnostic system.

## References

[1] Mahlknecht P, Kiechl S, Bloem BR, Willeit J, Scherfler C, Gasperi A, Rungger G, Poewe W, Seppi K (2013). Prevalence and burden of gait disorders in elderly men and women aged 60–97 years: a population-based study. PLoS One.

[2] Muro-de-la-Herran, A., Garcia-Zapirain, B., M´endez-Zorrilla, A.: Gait analysis methods: An overview of wearable and non-wearable systems, highlighting clinical applications. Sensors **14**(2), 3362–3394 (2014) 10.3390/s140203362 PMC3958266 24556672

[3] Lee, D.-W., Jun, K., Lee, S., Ko, J.-K., Kim, M.S.: Abnormal gait recognition using 3d joint information of multiple kinects system and rnn-lstm. In: 2019 41st Annual International Conference of the IEEE Engineering in Medicine and Biology Society (EMBC), pp. 542–545 (2019) 10.1109/EMBC.2019.8857607 31945957

[4] V´asquez-Correa, J.C., Arias-Vergara, T., Orozco-Arroyave, J.R., Eskofier, B., Klucken, J., Nöth, E.: Multimodal assessment of parkinson’s disease: A deep learning approach. IEEE Journal of Biomedical and Health Informatics **23**(4), 1618–1630 (2019) 10.1109/JBHI.2018.2866873 30137018

[5] Abtahi M, Bahram Borgheai S, Jafari R, Constant N, Diouf R, Shahriari Y, Mankodiya K (2020). Merging fnirs-eeg brain monitoring and body motion capture to distinguish parkinsons disease. IEEE Transactions on Neural Systems and Rehabilitation Engineering.

[6] Turner A, Hayes S (2019). The classification of minor gait alterations using wearable sensors and deep learning. IEEE Transactions on Biomedical Engineering.

[7] McCay KD, Hu P, Shum HPH, Woo WL, Marcroft C, Embleton ND, Munteanu A, Ho ESL (2022). A pose-based feature fusion and classification framework for the early prediction of cerebral palsy in infants. IEEE Transactions on Neural Systems and Rehabilitation Engineering.

[8] Aich, S., Pradhan, P.M., Park, J., Kim, H.-C.: A machine learning approach to distinguish parkinson’s disease (pd) patient’s with shuffling gait from older adults based on gait signals using 3d motion analysis. Int. J. Eng. Technol **7**(3.29), 153–156 (2018)

[9] Begg RK, Palaniswami M, Owen B (2005). Support vector machines for automated gait classification. IEEE Transactions on Biomedical Engineering.

[10] Chauhan, N.K., Singh, K.: A review on conventional machine learning vs deep learning. In: 2018 International Conference on Computing, Power and Communication Technologies (GUCON), pp. 347–352 (2018). 10.1109/GUCON.2018.8675097

[11] Ramachandran R, Rajeev D, Krishnan S, Subathra P (2015). Deep learning an overview. International Journal of Applied Engineering Research.

[12] Davarzani, S., Saucier, D., Peranich, P., Carroll, W., Turner, A., Parker, E., Middleton, C., Nguyen, P., Robertson, P., Smith, B., *et al.*: Closing the wearable gap—part vi: Human gait recognition using deep learning methodologies. Electronics **9**(5), 796 (2020)

[13] McCay KD, Ho ESL, Shum HPH, Fehringer G, Marcroft C, Embleton ND (2020). Abnormal infant movements classification with deep learning on pose-based features. IEEE Access.

[14] Ramzan F, Khan MUG, Rehmat MA, Iqbal S, Saba T, Rehman A, Mehmood Z (2020). A deep learning approach for automated diagnosis and multi-class classification of alzheimer’s disease stages using resting-state fmri and residual neural networks. J. Medical Syst..

[15] Karakus, B.A., Yildirim, O¨ ., Talo, M., Baloglu, U.B., Aydin, G.,Puthankattil, S.D., Acharya, U.R.: Automated depression detection using deep representation and sequence learning with EEG signals.J. Medical Syst. 43(7), 205–120512 (2019). 10.1007/s10916-019-1345-y 10.1007/s10916-019-1345-y 31139932

[16] Ganapathy, N., Veeranki, Y.R., Kumar, H., Swaminathan, R.: Emotion recognition using electrodermal activity signals and multiscale deep convolutional neural network. J. Medical Syst. **45**(4), 49 (2021). 10.1007/s10916-020-01676-6 10.1007/s10916-020-01676-6 33660087

[17] Shorten, C., Khoshgoftaar, T.M.: A survey on image data augmentation for deep learning. J. Big Data 6, 60 (2019). 10.1186/s40537-019-0197-0 10.1186/s40537-021-00492-0 PMC8287113 34306963

[18] Zhu, M., Men, Q., Ho, E.S.L., Leung, H., Shum, H.P.H.: Interpreting deep learning based cerebral palsy prediction with channel attention. In: 2021 IEEE EMBS International Conference on Biomedical and Health Informatics (BHI), pp. 1–4 (2021). 10.1109/BHI50953.2021.959508619

[19] Rueangsirarak W, Zhang J, Aslam N, Ho ESL, Shum HPH (2018). Automatic musculoskeletal and neurological disorder diagnosis with relative joint displacement from human gait. IEEE Transactions on Neural Systems and Rehabilitation Engineering.

[20] Analysis, M.: Leading the industry in optical motion capture solutions. http://www.motionanalysis.com (2017)

[21] Collins TD, Ghoussayni SN, Ewins DJ, Kent JA (2009). A six degrees-of-freedom marker set for gait analysis: repeatability and comparison with a modified helen hayes set. Gait & posture.

[22] Meijering E (2002). A chronology of interpolation: from ancient astronomy to modern signal and image processing. Proceedings of the IEEE.

[23] Saravanakumar, S., Thangaraj, P.: A computer aided diagnosis system for identifying alzheimer’s from MRI scan using improved adaboost. Medical Syst. 43(3), 76–1768 (2019). 10.1007/s10916-018-1147-7 10.1007/s10916-018-1147-7 30756191

[24] Acharya UR, Fernandes SL, Wei JKE, Ciaccio EJ, Fabell MKBM, Tanik UJ, Rajinikanth V, Yeong CH (2019). Automated detection of alzheimer’s disease using brain MRI images- A study with various feature extraction techniques. J. Medical Syst..

[25] Sakkos D, Mccay KD, Marcroft C, Embleton ND, Chattopadhyay S, Ho ESL (2021). Identification of abnormal movements in infants: A deep neural network for body part-based prediction of cerebral palsy. IEEE Access.

[26] Zhang, H., Cisse, M., Dauphin, Y.N., Lopez-Paz, D.: mixup: Beyond empirical risk minimization. arXiv preprint arXiv:1710.09412 (2017)

[27] Panwar, M., Biswas, D., Bajaj, H., Jöbges, M., Turk, R., Maharatna, K., Acharyya, A.: Rehab-net: Deep learning framework for arm movement classification using wearable sensors for stroke rehabilitation. IEEE Transactions on Biomedical Engineering **66**(11), 3026–3037 (2019) 10.1109/TBME.2019.2899927 30794162

[28] Chawla NV, Bowyer KW, Hall LO, Kegelmeyer WP (2002). Smote: synthetic minority over-sampling technique. Journal of artificial intelligence research.

[29] Boytim MJ, Fischer DA, Neumann L (1991). Syndesmotic ankle sprains. The American journal of sports medicine.

[30] Bos RA, Nizamis K, Koopman BFJM, Herder JL, Sartori M, Plettenburg DH (2020). A case study with symbihand: An semg-controlled electrohydraulic hand orthosis for individuals with duchenne muscular dystrophy. IEEE Transactions on Neural Systems and Rehabilitation Engineering.

[31] Mosh´e, S.L., Perucca, E., Ryvlin, P., Tomson, T.: Epilepsy: new advances. The Lancet **385**(9971), 884–898 (2015) 10.1016/S0140-6736(14)60456-6 25260236

[32] Roels J, Martens M, Mulier J, Burssens A (1978). Patellar tendinitis (jumper’s knee). The American journal of sports medicine.

[33] Lunn, M.R., Wang, C.H.: Spinal muscular atrophy. The Lancet (9630), 2120–2133 (2008) 10.1016/S0140-6736(08)60921-6 18572081

[34] Martinez-Murcia FJ, Ortiz A, Gorriz J-M, Ramirez J, Castillo-Barnes D (2020). Studying the manifold structure of alzheimer’s disease: A deep learning approach using convolutional autoencoders. IEEE Journal of Biomedical and Health Informatics.

[35] Kubota K, Hanawa H, Yokoyama M, Kita S, Hirata K, Fujino T, Kokubun T, Ishibashi T, Kanemura N (2021). Usefulness of muscle synergy analysis in individuals with knee osteoarthritis during gait. IEEE Transactions on Neural Systems and Rehabilitation Engineering.

[36] Enoka RM, Duchateau J (2008). Muscle fatigue: what, why and how it influences muscle function. The Journal of physiology.

[37] Xia Y, Yao Z, Ye Q, Cheng N (2020). A dual-modal attention-enhanced deep learning network for quantification of parkinson’s disease characteristics. IEEE Transactions on Neural Systems and Rehabilitation Engineering.

[38] Zhang, P., Lan, C., Zeng, W., Xing, J., Xue, J., Zheng, N.: Semantics-guided neural networks for efficient skeleton-based human action recognition. In: 2020 IEEE/CVF Conference on Computer Vision and Pattern Recognition (CVPR), pp. 1109–1118 (2020)

[39] Yoneyama M, Kurihara Y, Watanabe K, Mitoma H (2013). Accelerometry-based gait analysis and its application to parkinson’s disease assessment— part 2: A new measure for quantifying walking behavior. IEEE Transactions on Neural Systems and Rehabilitation Engineering.

[40] Rattani, A., Kisku, D.R., Bicego, M., Tistarelli, M.: Feature level fusion of face and fingerprint biometrics. In: 2007 First IEEE International Conference on Biometrics: Theory, Applications, and Systems, pp. 1–6 (2007)

[41] Gupta A, Jadhav A, Jadhav S, Thengade A, Iyer B, Rajurkar AM, Gudivada V (2020). Human gait analysis based on decision tree, random forest and knn algorithms. Applied Computer Vision and Image Processing.

[42] Devi Das, K., Saji, A.J., Kumar, C.S.: Frequency analysis of gait signals for detection of neurodegenerative diseases. In: 2017 International Conference on Circuit ,Power and Computing Technologies (ICCPCT), pp. 1–6 (2017). 10.1109/ICCPCT.2017.8074273

[43] Hu, J., Shen, L., Sun, G.: Squeeze-and-excitation networks. In: 2018 IEEE/CVF Conference on Computer Vision and Pattern Recognition (CVPR), pp. 7132–7141 (2018). 10.1109/CVPR.2018.00745

[44] Judge, J.O., King, M.B., Whipple, R., Clive, J., Wolf son, L.I.: Dynamic balance in older persons: effects of reduced visual and proprioceptive input. The Journals of Gerontology Series A: Biological Sciences and Medical Sciences **50**(5), 263–270 (1995) 10.1093/gerona/50a.5.m263 7671028

[45] Ishikawa G, Nagakura Y, Takeshita N, Shimizu Y (2014). Efficacy of drugs with different mechanisms of action in relieving spontaneous pain at rest and during movement in a rat model of osteoarthritis. European journal of pharmacology.

